# Genetic Polymorphism of Human Y Chromosome and Risk Factors for Cardiovascular Diseases: A Study in WOBASZ Cohort

**DOI:** 10.1371/journal.pone.0068155

**Published:** 2013-07-25

**Authors:** Grażyna Kostrzewa, Grażyna Broda, Magdalena Konarzewska, Paweł Krajewki, Rafał Płoski

**Affiliations:** 1 Department of Medical Genetics, Medical University of Warsaw, Warsaw, Poland; 2 Department of Cardiovascular Epidemiology and Prevention, and Health Promotion, Institute of Cardiology, Warsaw, Poland; 3 Department of Forensic Medicine, Medical University of Warsaw, Warsaw, Poland; Innsbruck Medical University, Austria

## Abstract

Genetic variants of Y chromosome predispose to hypertension in rodents, whereas in humans the evidence is conflicting. Our purpose was to study the distribution of a panel of Y chromosome markers in a cohort from a cross-sectional population-based study on the prevalence of cardiovascular risk factors in Poland (WOBASZ study). The HindIII, YAP Y chromosome variants, previously shown to influence blood pressure, lipid traits or height, as well as SNPs defining main Y chromosome haplogroups, were typed in 3026, 2783 and 2652 samples, respectively. In addition, 4 subgroups (N∼100 each) representing extremes of LDL concentration or blood pressure (BP) were typed for a panel of 17 STRs. The HindIII and YAP polymorphism were not associated with any of the studied traits. Analysis of the haplogroup distribution showed an association between higher HDL level and hg I-M170 (P = 0.02), higher LDL level and hg F*(xI-M170, J2-M172, K-M9) (P = 0.03) and lower BMI and hg N3-Tat (P = 0.04). Analysis of STRs did not show statistically significant differences. Since all these associations lost statistical significance after Bonferroni correction, we conclude that a major role of Y chromosome genetic variation (defined by HindIII, YAP or main Y chromosome haplogroups) in determining cardiovascular risk in Poles is unlikely.

## Introduction

Genetic variants of Y chromosome have been shown to predispose to hypertension in a series of rodent experiments [1]–[3] with a recent suggestion of direct involvement of variation in the SRY locus [4], [5]. In humans, it has been found that a hypertensive father, but not a hypertensive mother, determines blood pressure in male offspring [6]. Furthermore, a variant of Y human chromosome defined by HindIII polymorphism in the centromeric region has been associated with hypertension in males from the general population of Australia [7], Scotland, Poland [8], [9] and the US [10], whereas in Spain an association limited to myocardial infarction patients was found [11]. A large study in a Polish population reported that in addition to its effect on blood pressure the HindIII variant also influenced cholesterol concentration, indicating a potential broad role of Y chromosome polymorphism in determining cardiovascular risk [9]. Consistent with this, trends for association between blood pressure/lipid profile and Y chromosome variants were observed among the Japanese, although here the associated variants were defined by YAP polymorphism (an Alu insertion) [12], [13].

Despite the abovementioned reports, the association between Y chromosome HindIII polymorphism and hypertension or lipid profile remains controversial as it was not observed in recent relatively large studies encompassing Caucasians from the UK [14], [15], Belgium or Italy [14]. Lack of effect of HindIII Y chromosome variants on cardiovascular risk factors was also reported in another recent study in Caucasians (UK, Italy) and South Asians (UK) although in the same study a haplotype defined by other markers (rs768983 and rs3212292) was linked with a favorable lipoprotein pattern in Blacs [16].

Whereas analysis of candidate markers chosen on the basis of proven or likely functional significance represent a direct way to study the role of Y chromosome variation in predisposing to disease(s), the problem may also be approached by taking advantage of growing knowledge of the evolutionary history of this part of the genome. Since the major part of Y chromosome does not recombine during meiosis, all newly arising variants remain in virtually absolute linkage disequilibrium with variants present in the chromosome in which the mutation has occurred [14]. Thus, a substantial part of Y chromosome variability may be captured by relatively few markers, provided they identify major evolutionary branches of Y chromosomes present in a population (Y chromosome haplogroups-YHg). Recently we analyzed distribution of YHgs in a Polish population identifying the most frequent variants as R1a1 -M17, R1* (xR1a1-M17), I -M170, E3b -M35, J2 -M172, N3 -Tat [15].

In order to resolve the described controversies we set out to study the distribution of Y chromosome genetic markers in a large sample of Polish males, comprehensively characterized with respect to cardiovascular risk factors and ascertained independently from previously studied cohorts [8], [9]. The studied markers included the HindIII and YAP polymorphisms as well as a panel of SNP allowing assignment of major YHgs found in the Polish population and a panel of hypervariable STRs.

## Materials and Methods

### Subjects

We studied males from the WOBASZ study, which is a cross-sectional population-based study aimed at delineation of classical and genetic risk factors for cardiovascular diseases as described previously [16]–[18]. The WOBASZ participants were randomly chosen from the Polish population register of permanent residents aged 20–74 years. The study comprised the whole territory of Poland and was performed in cooperation with six regional medical research centers: Institute of Cardiology in Warsaw, Medical University in Lodz, Medical University in Poznan, Silesian Medical University in Katowice and Jagiellonian University in Krakow. The sampling scheme was composed of sex, administration units and type of urban development. Data were collected from January 2003 to December 2005 and the response rate was 70%.

All participants gave two consents in writing: one for evaluation of conventional cardiovascular risk factors and one for taking a blood sample for DNA isolation and genetic tests. Questionnaire data were collected by certified pollsters who were also responsible for ascertaining that all subjects had full capacity/ability to participate in the study as well as to give their informed consent. The study was approved by The Medical Ethics Committee of the National Institute of Cardiology in Warsaw.

The height and weight of each patient were measured and the body mass index (BMI) was calculated according to the formula: BMI = weight (kg)/height (m)^2^. Blood pressure measurements were taken thrice on the right arm after 5 minutes of rest in a sitting position in one minute intervals. The value used in analyses was the average of the 2nd and 3rd measurement. Hypertension was defined as systolic blood pressure of 140 mm Hg or higher, a diastolic blood pressure of 90 mm Hg or higher, or use of antihypertensive medication.

Two blood samples obtained after 8–12 h of fasting were used for DNA isolation and for biochemistry tests, respectively. The blood samples for DNA extraction were stored at −70°C until the DNA was isolated by a salting out-method. Biochemical tests (cholesterol, triglycerides and glucose) were performed at the Central Laboratory of the National Institute of Cardiology in Warsaw. Total cholesterol, LDL-cholesterol, HDL-cholesterol and glucose concentration were estimated using the Roche INTEGRA 400 Analyzer with Roche reagents. Concentration of triglycerides (TG) was determined by the enzymatic-colorimetric method (GPO/PAP/kit), with glycerophosphate and 4-amidoantypiryn. Glucose concentration was estimated using the enzymatic reference method with hexokinase.

In males typed for HindIII from whom a sufficient amount of DNA was available, we also performed an analysis of YAP variants and Y chromosome haplogroups. The call rate was >0.95% and the results were obtained for following numbers of samples: N = 3026 (HindIII), N = 2 783 (YAP) and N = 2 652 (Y chr. haplogroups). Furthermore, we performed an analysis of STR markers in four groups of ∼100 samples representing extreme phenotypes for blood pressure and LDL concentration, which are likely to be enriched for genetic variants influencing these traits. The groups were selected from those genotyped for HindIII polymorphism according to following criteria: group 1 (H_BP) – presence of hypertension, age below 35 years, group 2 (L_BP) – normal blood pressure, age over 55 years, group 3 (H_LDL) – LDL concentration above 4 mmol/l, age below 40 years, group 4 (L_LDL) – LDL concentration below 3 mmol/l, age over 55 years. The call rate in STR analysis ranged from >0.92%–98% (File S1).

### Genotyping

Genotyping for HindIII polymorphism was performed by PCR-RFLP as described previously [7]. Typing for YAP polymorphism was performed according to Hammer et al. [19].

**Figure 1:**
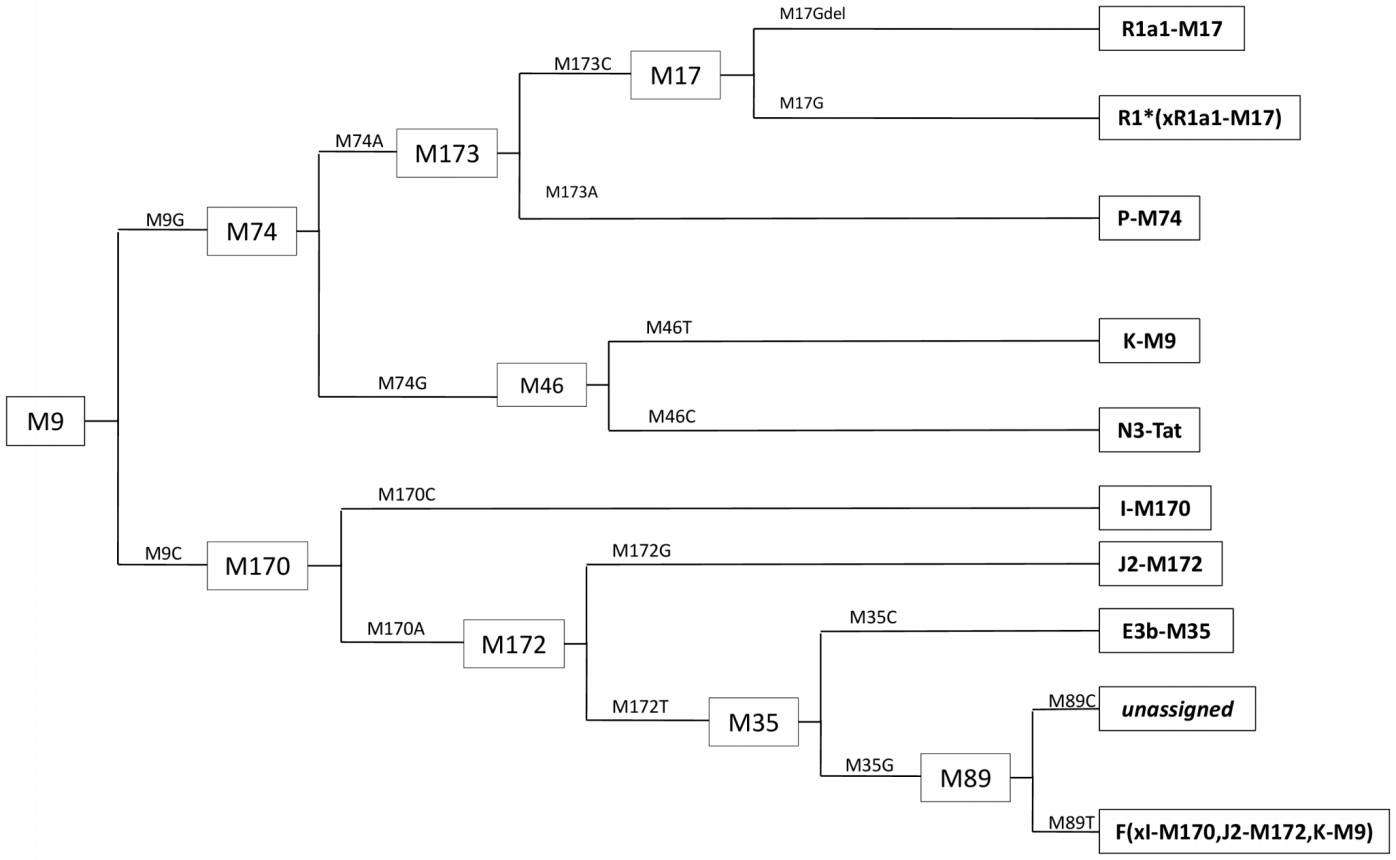
Scheme of the haplogroup typing based on a phylogenetic tree published by YCC [20].

Haplogroup typing was based on a phylogenetic tree published by YCC [20] and was performed in several stages as illustrated in figure 1. In the first stage the M9 marker was typed using PCR primers described previously [21]. PCR was performed in a 10 µl reaction volume (detailed composition of the PCR mixture and thermal conditions indicated in tab 1). Eight microliters of the PCR products were digested with 2.5 U of HinfI (Fermentas, Vilnius, Lithuania) at 37°C, and the digestion products were analyzed in a 3% agarose gel using staining with ethidium bromide. The mutated (G) allele yielded a 164 bp product, whereas the wild type variant (C) yielded products of 100 bp and 64 bp.

**Table 1:** Summary table of all tested markers, PCR primers sequences with their final concentrations, composition of the PCR mixtures and thermal conditions of the amplification.

SNP	RefSNP ID	PCR primers sequence	primers (pM)	PCR Mix	PCR conditions	PCR size (bp)	SNaPshot primers sequence
HindIII	rs768983	Ellis et al. [7]				285	
YAP	-	Hammer et al. [19]	7			150/455	
M9	rs3900	Kayser et al. [15]	7	2 mM of each dNTP (Roche), 3 mM MgCl_2_, 2,5 U of Taq DNA Polymerase (Fermetas) and 75 ng of template	95°C for 4 min, (95°C for 30 s, 54°C for 30 s, and 72°C for 45 s)*45, final extension of 7 min at 72°C	164	
M17*	rs3908	F: 5′-CCT GGT CAT AAC ACT GGA AAT C-3′	3			170	1 5′- GTCGTGAAAGTCTGACAACCAAAATTCACTTAAAAAAACCC-3′
		R: 5′-AGC TGA CCA CAA ACT GAT GTA GA-3′					2 5′–CCA GAG TTT GTG GTT GCT GGT TGT TAC GGG-3′
M173*	rs2032624	F: 5′-TTT TCT TAC AAT TCA AGG GCA TTT AG-3′	10			81	5′-AGT CTG ACA ATA CAA TTC AAG GGC ATT TAG AAC-3′
		R: 5′-CTG AAA ACA AAA CAC TGG CTT ATC A-3′					
Tat(M46)*	rs34442126	F: 5′-TAT ATG GAC TCT GAG TGT AGA CTT GTG A-3′	20	10 mM of each dNTP, (Roche), 5 mM MgCl_2_ 1,5 U Taq DNA Polymerase (Fermentas) and 100 ng of template	95°C for 5 min, (95°C for 30 s, 60°C for 55 s, and 72°C for 60 s)*35 final extension of 7 min at 72°C	115	5′-AAG TCT GAC AAG CTC TGA AAT ATT AAA TTA AAA CAA C-3′
		R: 5′-GGT GCC GTA AAA GTG TGA AAT AAT C-3′					
M170**	rs2032597	F: 5′-CAG CTC TTA TTA AGT TAT GTT TTC ATA TTC TGT G-3′	3,5			119	5′-AAG TCT GAC AAC AAC CCA CAC TGA AAA AAA-3′
		R: 5′-GTC CTC ATT TTA CAG TGA GAC ACA AC-3′					
M172**	rs2032604	F: 5′-TGA GCC CTC TCC ATC AGA AG-3′	21			179	5′-TGA AAG TCT GAC AAC AAA CCC ATT TTG ATG CTT-3′
		R: 5′-GCC AGG TAC AGA GAA AGT TTG G-3′					
M35**	-	F: 5′-AGG GCA TGG TCC CTT TCT AT-3′	8			96	5′-CGT CGT GAA AGT CTG ACA ATC GGA GTC TCT GCC TGT GTC-3′
		R: 5′-TCC ATG CAG ACT TTC GGA GT-3′					
M74	rs2032635	F: 5′-AAC TAG GAA AGT CTG AAA AAT AAT CAG A-3′	7	2 mM of each dNTP (Roche), 3 mM MgCl_2_, 2,5 U of Taq DNA Polymerase (Fermetas) and 75 ng of template	95°C for 5 min, (95°C for 30 s, 56°C for 30 s, and 72°C for 40 s)*30, final extension of 5 min at 72°C	151	
		R: 5′- GCT GCT GTT GTC TTT TAA GTA ACT TAC T-3′					
M89	rs2032652	Ke et al. [23]	5			87	

*Triplex 1/** Triplex 2.

Samples typed as M9G were subsequently genotyped by SNaPshot technique for the M17, M173 and Tat (M46) markers, whereas samples typed as M9C for the M170, M172 and M35 markers. PCR amplification of these markers was performed in two triplex reactions. Primers were designed according to guidelines of Juan and Sanchez [22]. Their final concentration in the reaction mix, the composition of the PCR mixture and thermal conditions are summarized in tab 1. PCR products were treated with exonuclease I and shrimp alkaline phosphatase (Exo-Sap Fermentas) and 3 µl of the product was used in a SNapShot reaction with the primers summarized in table 1. Primers' concentration and reaction conditions were maintained according to the manufacturer's instructions (*Applied Biosystems*, *Foster City*, *CA*). After the SNaPshot reaction samples were run on a *Genetic Analyzer 3130* (Applied Biosystems) and the data were analyzed by *GeneScan* Analysis software (*Applied Biosystems*). Analysis of chromatograms for each triplex allowed to assign samples to haplogroups: R1a1-M17, R1*(xR1a1-M17), J2-M172, N3-Tat, I-M170 and E3b-M35. Samples with no mutated allele in any of the tested loci were additionally typed for M74 and M89 markers. PCR amplification of the M74 marker was performed according to data shown in Tab 1. Seven microliters of the PCR products were digested with 4 U of RsaI (*Fermentas*, *Vilnus*, *Lithuania*) at 37°C for 3 hours. RsaI digestion yields fragments of sizes 104 and 47 bp when the segment contains allele M74A and 151 bp when the probe contains ancestral allel M74G. Typing for M89 marker was performed according to Ke et al. [23]. Y chromosome STR analysis including the following loci: DYS456, DYS3891, DYS390, DYS3892, DYS458, DYS19, DYS393, DYS391, DYS439, DYS635, DYS392, GATAH4, DYS437 and DYS438 was performed using *Y-Filer* kit from *Applied Biosystems*, according to the manufacturer's instructions. DYS385 1 and DYS385 2 were not analyzed due to the fact that these loci are amplified by one set of Y-filer primers and thus cannot be reliably distinguished [24].

### Statistical analysis

For continuous variables, differences between groups were analyzed with student t test. Calculations were performed with *Statistica* or *SPSS* software packages. In the analysis of the Y chromosome haplogroups, Bonferroni correction was applied with correction factor of 7 (number of haplogroups analyzed).

Our study had the following power to detect effects of HindIII polymorphism, as previously reported in a Polish population [8], [9]: diastolic BP – increase of 2.6 mmHg, power = 0.9998; systolic BP – increase of 5.27 mmHg, power = 0.9999; total cholesterol – decrease of 0.15 mmol (i.e. 5.8 mg%), power = 0.90; LDL cholesterol – decrease of 0.15 mmol (i.e. 5.8 mg%), power = 0.96.

## Results

### Analysis of HindIII and YAP polymorphisms

Distribution of the studied parameters according to Y chromosome HindIII and YAP markers is shown in Table 1. There are no statistically significant differences in any of the comparisons. We noted that both mean systolic and diastolic blood pressure showed trends for lower values among males with HindIII (+) genotype vs. those with HindIII (−) (Table 2).

**Table 2:** Distribution of studied parameters according to Y chromosome HindIII and YAP markers.

	HindIII (−)			HindIII (+)				YAP (−)			YAP (+)			
	N	Mean	SD	N	Mean	SD	p	N	Mean	SD	N	Mean	SD	p
**Total cholesterol** (mmol/l)	2212	5.4	1.1	801	5.4	1.1	0.68	2676	5.4	1.1	96	5.4	1.2	0.67
**LDL cholesterol** (mmol/l)	2166	3.3	1.0	793	3.3	1.0	0.49	2626	3.3	1.0	96	3.3	1.0	0.77
**HDL cholesterol** (mmol/l)	2211	52.1	15.3	802	53.0	17.1	0.19	2675	52.2	15.6	96	52.6	17.6	0.81
**BP systolic**	2217	138.2	18.5	808	137.3	18.3	0.25	2686	138.0	18.5	96	136.2	20.5	0.36
**BP diastolic**	2217	83.9	11.6	808	83.7	11.4	0.58	2686	83.8	11.5	96	82.8	11.9	0.38
**TG** (mmol/l)	2212	1.7	1.5	802	1.7	1.4	0.51	2676	1.7	1.4	96	1.7	1.5	0.95
**BMI**	2208	26.8	4.6	807	26.6	4.3	0.38	2676	26.8	4.5	96	26.5	4.2	0.60
**Age (years)**	2218	45.8	14.9	808	46.1	15.1	0.65	2687	45.9	14.9	96	46.6	16.1	0.68
**Height (cm)**	2212	174.4	7.0	807	174.4	7.0	0.79	2680	174.4	7.0	96	174.0	7.2	0.57
**Weigh (kg)**	2211	81.6	14.8	808	81.0	14.0	0.29	2680	81.5	14.7	96	80.3	13.7	0.45
**Waist (cm)**	2217	95.7	12.4	808	95.3	12.1	0.54	2686	95.6	12.4	96	96.2	11.7	0.63
**Hips circuference** (cm)	2209	103.8	8.6	806	103.8	8.1	0.83	2676	103.8	8.5	96	103.4	7.8	0.69
**Glucose** (mg%)	2206	93.1	26.5	801	92.2	26.9	0.43	2670	93.1	26.8	95	90.2	16.7	0.30

### Analysis of Y chromosome haplogroups

In Table 3 distribution of studied parameters according to Y chromosome haplogroup is shown. We observed three statistically significant associations at alpha = 0.05. Among males with haplogroup I-M170 mean HDL level (53.8 mmol/l, SD = 17.4, N = 449) was higher than among those with other haplogroups (51.7 mmol/l, SD = 15.2, N = 2195, P = 0.02). Among males with haplogroup F*(xI-M170, J2-M172,K-M9) mean LDL level (3.6 mmol/l, SD = 1.1, N = 54) was higher than among those with other haplogroups (3.3 mmol/l, SD = 0.95, N = 2538, P = 0.03). Among males with haplogroup N3-Tat mean BMI (25.8, SD = 3.9, N = 86) was lower than among those with other haplogroups (26.6, SD = 4.3, N = 2559, P = 0,04). None of these associations remained statistically significant after Bonferroni correction.

**Table 3:** Distribution of studied parameters according to Y chromosome haplogroup (only haplogroups with n>10 were analyzed).

	R1a1*			I*			R1*			E3b*			F*			J2*			N3*		
	(N = 1493–1528)			(N = 446–451)			(N = 340–355)			(N = 97)			(N = 54–57)			(N = 63–65)			(N = 83–86)		
	M	SD	P	M	SD	P	M	SD	P	M	SD	P	M	SD	P	M	SD	P	M	SD	P
TC (mmol/l)	5.4	1.1	0.41	5.5	1.1	0.16	5.4	1.1	0.57	5.3	1.2	0.34	5.6	1.3	0.19	5.3	1.1	0.31	5.3	1.0	0.64
LDL (mmol/l)	3.3	0.9	0.86	3.4	0.9	0.31	3.3	1.0	0.48	3.2	1.0	0.37	3.6	1.1	**0.03**	3.2	1.0	0.56	3.3	0.9	0.89
HDL (mmol/l)	51.7	15.0	0.09	53.8	17.4	**0.02**	52.2	15.5	0.96	53.3	18.8	0.46	50.1	11.9	0.32	51.6	15.3	0.76	52.4	17.5	0.89
BP sys. (mmHg)	137.8	18.1	0.60	137.3	17.8	0.41	139.5	20.3	0.11	136.3	20.5	0.35	136.4	20.0	0.51	136.7	19.6	0.56	140.8	17.9	0.15
BP dias. (mmHg)	83.6	11.4	0.29	83.9	11.7	0.88	84.7	12.0	0.13	82.7	11.8	0.29	83.9	10.9	0.97	83.0	11.7	0.53	84.8	12.0	0.42
TG (mmol/l)	1.7	1.5	0.85	1.6	1.1	0.30	1.8	1.7	0.11	1.7	1.5	0.69	1.8	1.3	0.76	1.6	1.0	0.38	1.8	2.6	0.46
BMI	26.8	4.6	0.63	26.7	4.3	0.90	27.0	4.7	0.17	26.3	4.1	0.36	26.6	4.2	0.79	26.2	4.2	0.36	25.8	3.9	**0.04**
Age (ys.)	45.8	14.9	0.57	46.3	15.0	0.60	46.4	14.7	0.53	46.3	16.1	0.84	44.5	14.6	0.43	44.0	15.7	0.28	45.7	15.5	0.84
Height(cm)	174.4	7.2	0.58	173.9	6.8	0.21	174.1	7.0	0.66	174.0	7.4	0.68	174.7	6.8	0.71	175.7	5.8	0.10	175.3	7.0	0.22
Weigh kg)	81.5	15.2	0.38	80.8	13.7	0.42	82.0	15.4	0.32	79.8	13.3	0.29	80.8	11.3	0.78	81.2	14.6	0.94	79.3	13.8	0.19
Waist (cm)	95.6	12.6	0.83	95.4	12.0	0.77	96.0	12.8	0.52	95.9	11.5	0.80	95.7	11.1	0.93	94.6	12.1	0.56	93.9	12.1	0.21
Hips circ. (cm)	103.8	8.6	0.69	103.6	8.1	0.63	104.0	9.1	0.61	103.5	7.8	0.73	104.2	7.2	0.72	103.4	7.5	0.70	102.7	7.2	0.26
Glucose (mg%)	92.3	24.8	0.23	92.9	27.8	0.94	95.1	31.7	0.09	90.4	22.6	0.35	93.4	28.6	0.88	95.1	35.2	0.50	91.1	21.9	0.53

TC –total cholesterol, M – Mean, SD standard deviation, P - p value (t-test vs. all other haplogroups), BP blood pressure, TG-triglycerides

### Analysis of Y chromosome STRs

When comparing distribution of alleles of 19 Y chromosome STRs in groups with high and low LDL concentration (LDL_H, LDL_L, respectively), and high and low blood pressure (BP_H, BP_L, respectively) we found no statistically significant differences (Tables S5 and S16 in File S1).

## Discussion

While studying a relatively large population- based sample of Polish Caucasian males we did not observe an effect of the Y chromosome Hind III or YAP polymorphism on blood pressure, cholesterol concentration or other anthropological or biochemical parameters.

The main finding from our study is the lack of effect of HindIII variants on blood pressure. On one hand, among the associations between Y chromosome markers and cardiovascular risk factors studied so far, the link between HindIII polymorphism and hypertension has been most consistent with five studies reporting an effect [7]–[11]. On the other hand, this association was not replicated in recent studies in populations from the UK, Belgium and Italy [25]–[27]. Whereas these discrepancies could in theory be attributed to population differences, our results make it unlikely, since we studied essentially the same population, i.e. Polish Caucasians, as did Charkar et al., who provided particularly strong support for the effect [8], [9]. It should be emphasized that the Polish population is homogenous with respect to Y chromosome markers, suggesting that the discrepancies are not caused by population stratification within Poland either [15], [28]. Lack of a genuine effect of HindIII polymorphism on blood pressure is also consistent with opposite directions of associations among studies which reported positive findings - in two studies the HindIII (+) variant was linked with lower BP [7], [10] whereas in the remaining studies the opposite was found [8], [9], [11].

Similarly as with BP, we did not observe an effect of HindIII polymorphism on cholesterol concentration in contrast to a previous study performed among Polish males [9] but in agreement with recent reports from other populations [25]–[27].

Our data do not support a role of HindIII variant in determining height, in agreement with Weedeon et al. [29] but contrary to the original observation by Ellis et al, [30]. Our study also does not provide evidence for an effect of YAP polymorphism, contrary to findings of Shoji et al. [12] but confirming conclusions of Hiura et al. who attributed observed trends for association between this Y chromosome variant and blood pressure as well as HDL concentration to chance [13].

In a substantial subgroup of males (N = 2652) we also determined distribution of SNPs allowing to assign major Y chromosome haplogroups. Whereas these SNPs probably do not have any functional significance, they define phylogenetically distinct Y chromosome pools and at least some of them would be expected to be in linkage disequilibrium with the putative variant(s) influencing cardiovascular risk sought in this study. Although we observed three associations significant at alpha = 0.05 we think they are unlikely to be genuine for two reasons: (i) none withstood Bonferroni correction despite the modest correction factor applied (n = 7), (ii) they did not point to a single haplogroup as harboring risk variant(s). Analysis of Y chromosome STRs in subgroups likely to represent the extremes of distribution of the genetic burden for high LDL concentration or high BP did not show any associations, further strengthening the conclusions from HindIII, YAP and haplogroups analyses.

In conclusion, our study shows that in humans Y chromosome genetic polymorphism, in particular the widely studied HindIII variant, does not influence blood pressure, lipid traits or anthropometric measures, such as height, weight or waist/hips circum ference.

## Supporting Information

File S1 **Comparison of distribution of alleles of studied Y-STR loci in groups with high and low LDL concentration (LDL_H, LDL_L, respectively) and high and low blood pressure (BP_H, BP_L, respectively).** P values calculated by permutation based Fisher exact test (10000 permutations, performed using SPSS). The ‘Total’ refers to samples in which result was obtained (initially 105 samples were selected for analysis). Table S1, Comparison of distribution of DYS456 in groups with high and low LDL concentration. Table S2, Comparison of distribution of DYS389I in groups with high and low LDL concentration. Table S3, Comparison of distribution of DYS390 in groups with high and low LDL concentration. Table S4, Comparison of distribution of DYS389II in groups with high and low LDL concentration. Table S5, Comparison of distribution of DYS19 in groups with high and low LDL concentration. Table S6, Comparison of distribution of DYS391 in groups with high and low LDL concentration. Table S7, Comparison of distribution of DYS439 in groups with high and low LDL concentration. Table S8, Comparison of distribution of DYS635 in groups with high and low LDL concentration. Table S9, Comparison of distribution of DYS392 in groups with high and low LDL concentration. Table S10, Comparison of distribution of GATAH4 in groups with high and low LDL concentration. Table S11, Comparison of distribution of DYS437 in groups with high and low LDL concentration. Table S12, Comparison of distribution of DYS448 in groups with high and low LDL concentration. Table S13, Comparison of distribution of DYS456 in groups with high and low blood pressure. Table S14, Comparison of distribution of DYS389I in groups with high and low blood pressure. Table S15, Comparison of distribution of DYS389II in groups with high and low blood pressure. Table S16, Comparison of distribution of DYS19 in groups with high and low blood pressure. Table S17, Comparison of distribution of DYS385I in groups with high and low blood pressure. Table S18, Comparison of distribution of DYS391 in groups with high and low blood pressure. Table S19, Comparison of distribution of DYS439 in groups with high and low blood pressure. Table S20, Comparison of distribution of DYS635 in groups with high and low blood pressure. Table S21, Comparison of distribution of GATAH4 in groups with high and low blood pressure. Table S22, Comparison of distribution of DYS437 in groups with high and low blood pressure. Table S23, Comparison of distribution of DYS438 in groups with high and low blood pressure. Table S24, Comparison of distribution of DYS448 in groups with high and low blood pressure.
(DOC)
