# Supplementary material for: Genetic Polymorphism of Human Y Chromosome and Risk Factors for Cardiovascular Diseases: A Study in WOBASZ Cohort
Source: PLoS One. 2013 Jul 25;8(7):e68155. doi: 10.1371/journal.pone.0068155 (PMC3723826; doi:10.1371/journal.pone.0068155)
Supplement: File S1 — Comparison of distribution of alleles of studied Y-STR loci in groups with high and low LDL concentration (LDL_H, LDL_L, respectively) and high and low blood pressure (BP_H, BP_L, respectively). P values calculated by permutation based Fisher exact test (10000 permutations, performed using SPSS). The ‘Total’ refers to samples in which result was obtained (initially 105 samples were selected for analysis). Table S1, Comparison of distribution of DYS456 in groups with high and low LDL concentration. Table S2, Comparison of distribution of DYS389I in groups with high and low LDL concentration. Table S3, Comparison of distribution of DYS390 in groups with high and low LDL concentration. Table S4, Comparison of distribution of DYS389II in groups with high and low LDL concentration. Table S5, Comparison of distribution of DYS19 in groups with high and low LDL concentration. Table S6, Comparison of distribution of DYS391 in groups with high and low LDL concentration. Table S7, Comparison of distribution of DYS439 in groups with high and low LDL concentration. Table S8, Comparison of distribution of DYS635 in groups with high and low LDL concentration. Table S9, Comparison of distribution of DYS392 in groups with high and low LDL concentration. Table S10, Comparison of distribution of GATAH4 in groups with high and low LDL concentration. Table S11, Comparison of distribution of DYS437 in groups with high and low LDL concentration. Table S12, Comparison of distribution of DYS448 in groups with high and low LDL concentration. Table S13, Comparison of distribution of DYS456 in groups with high and low blood pressure. Table S14, Comparison of distribution of DYS389I in groups with high and low blood pressure. Table S15, Comparison of distribution of DYS389II in groups with high and low blood pressure. Table S16, Comparison of distribution of DYS19 in groups with high and low blood pressure. Table S17, Comparison of distribution of DYS385I in groups with high and low blood press [file pone.0068155.s001.doc]

**Supplemental tables**

Table S1

|  |  | DYS 456 |  |  |  |  |  | Total |
| --- | --- | --- | --- | --- | --- | --- | --- | --- |
|  |  | 13 | 14 | 15 | 16 | 17 | 18 |  |
| LDL_H | n | 3 | 15 | 37 | 23 | 16 | 3 | 97 |
|  | % | 3.1 | 15.5 | 38.1 | 23.7 | 16.5 | 3.1 | 100 |
| LDL_L | n | 4 | 9 | 33 | 37 | 18 | 2 | 103 |
|  | % | 3.9 | 8.7 | 32.0 | 35.9 | 17.5 | 1.9 | 100 |

P=0.4

Table S2

|  |  | DYS 389I |  |  | Total |
| --- | --- | --- | --- | --- | --- |
|  |  | 12 | 13 | 14 |  |
| LDL_H | n | 17 | 70 | 12 | 99 |
|  | % | 17.2 | 70.7 | 12.1 | 100 |
| LDL_L | n | 13 | 80 | 10 | 103 |
|  | % | 12.6 | 77.7 | 9.7 | 100 |

P=0.5

Table S3

|  |  | DYS 390 |  |  |  |  |  |  |  |  | Total |
| --- | --- | --- | --- | --- | --- | --- | --- | --- | --- | --- | --- |
|  |  | 2 | 19 | 21 | 22 | 23 | 24 | 25 | 26 | 27 |  |
| LDL_H | n | 0 | 1 | 0 | 11 | 15 | 28 | 38 | 5 | 1 | 99 |
|  | % | 0 | 1.0 | 0.0 | 11.1 | 15.2 | 28.3 | 38.4 | 5.1 | 1.0 | 100 |
| LDL_L | n | 1 | 0 | 1 | 5 | 11 | 33 | 48 | 4 | 0 | 103 |
|  | % | 1.0 | 0.0 | 1.0 | 4.9 | 10.7 | 32.0 | 46.6 | 3.9 | 0.0 | 100 |

P=0.35

Table S4

|  |  | DYS 389II |  |  |  |  |  |  | Total |
| --- | --- | --- | --- | --- | --- | --- | --- | --- | --- |
|  |  | 27 | 28 | 29 | 30 | 31 | 32 | 33 |  |
| LDL_H | n | 2 | 12 | 29 | 33 | 14 | 9 | 0 | 99 |
|  | % | 2.0 | 12.1 | 29.3 | 33.3 | 14.1 | 9.1 | 0.0 | 100 |
| LDL_L | n | 0 | 10 | 29 | 39 | 17 | 4 | 1 | 100 |
|  | % | 0.0 | 10.0 | 29.0 | 39.0 | 17.0 | 4.0 | 1.0 | 100 |

P=0.5

Table S5

|  |  | DYS 19 |  |  |  |  |  | Total |
| --- | --- | --- | --- | --- | --- | --- | --- | --- |
|  |  | 13 | 14 | 15 | 16 | 17 | 18 |  |
| LDL_H | n | 3 | 24 | 16 | 39 | 17 | 0 | 99 |
|  | % | 3.0 | 24.2 | 16.2 | 39.4 | 17.2 | 0.0 | 100 |
| LDL_L | n | 6 | 20 | 16 | 32 | 25 | 2 | 101 |
|  | % | 5.9 | 19.8 | 15.8 | 31.7 | 24.8 | 2.0 | 100 |

P=0.4

Table S6

|  |  | DYS 391 |  |  |  |  |  | Total |
| --- | --- | --- | --- | --- | --- | --- | --- | --- |
|  |  | 1 | 9 | 10 | 11 | 12 | 13 |  |
| LDL_H | n | 0 | 0 | 58 | 39 | 2 | 1 | 100 |
|  | % | 0.0 | 0.0 | 58.0 | 39.0 | 2.0 | 1.0 | 100 |
| LDL_L | n | 1 | 1 | 65 | 33 | 3 | 0 | 103 |
|  | % | 1.0 | 1.0 | 63.1 | 32.0 | 2.9 | 0.0 | 100 |
| P=0.6 |  |  |  |  |  |  |  |  |

P=0.6

Table S7

|  |  | DYS 439 | |  |  |  |  | Total |
| --- | --- | --- | --- | --- | --- | --- | --- | --- |
|  |  | 8 | 10 | 11 | 12 | 13 | 14 |  |
| LDL_H | n | 0 | 30 | 35 | 20 | 14 | 1 | 100 |
|  | % | 0.0 | 30.0 | 35.0 | 20.0 | 14.0 | 1.0 | 100 |
| LDL_L | n | 1 | 37 | 29 | 22 | 10 | 2 | 101 |
|  | % | 1.0 | 36.6 | 28.7 | 21.8 | 9.9 | 2.0 | 100 |

P=0.7

Table S8

|  |  | DYS 635 |  |  |  |  |  | Total |
| --- | --- | --- | --- | --- | --- | --- | --- | --- |
|  |  | 20 | 21 | 22 | 23 | 24 | 25 |  |
| LDL_H | n | 1 | 10 | 13 | 66 | 6 | 4 | 100 |
|  | % | 1.0 | 10.0 | 13.0 | 66.0 | 6.0 | 4.0 | 100 |
| LDL_L | n | 0 | 12 | 11 | 62 | 13 | 4 | 102 |
|  | % | 0.0 | 11.8 | 10.8 | 60.8 | 12.7 | 3.9 | 100 |

P=0.5

Table S9

|  |  | DYS 392 |  |  |  |  |  |  |  | Total |
| --- | --- | --- | --- | --- | --- | --- | --- | --- | --- | --- |
|  |  | 0 | 8 | 10 | 11 | 12 | 13 | 14 | 15 |  |
| LDL_H | n | 0 | 1 | 0 | 83 | 1 | 11 | 4 | 0 | 100 |
|  | % | 0.0 | 1.0 | 0.0 | 83.0 | 1.0 | 11.0 | 4.0 | 0.0 | 100 |
| LDL_L | n | 2 | 0 | 1 | 76 | 3 | 14 | 3 | 2 | 101 |
|  | % | 2.0 | 0.0 | 1.0 | 75.2 | 3.0 | 13.9 | 3.0 | 2.0 | 100 |

P=0.4

Table S10

|  |  | GATA H4 |  |  |  |  | Total |
| --- | --- | --- | --- | --- | --- | --- | --- |
|  |  | 3 | 10 | 11 | 12 | 13 |  |
| LDL_H | n | 0 | 1 | 28 | 56 | 14 | 99 |
|  | % | 0.0 | 1.0 | 28.3 | 56.6 | 14.1 | 100 |
| LDL_L | n | 1 | 4 | 23 | 59 | 13 | 100 |
|  | % | 1.0 | 4.0 | 23.0 | 59.0 | 13 | 100 |

P=0.5

Table S11

|  |  | DYS 437 |  |  |  | Total |
| --- | --- | --- | --- | --- | --- | --- |
|  |  | 13 | 14 | 15 | 16 |  |
| LDL_H | n | 0 | 64 | 20 | 15 | 99 |
|  | % | 0.0 | 64.6 | 20.2 | 15.2 | 100 |
| LDL_L | n | 1 | 72 | 20 | 10 | 103 |
|  | % | 1.0 | 69.9 | 19.4 | 9.7 | 100 |

P=0.5

Table S12

|  |  | DYS 448 |  |  |  |  | Total |
| --- | --- | --- | --- | --- | --- | --- | --- |
|  |  | 18 | 19 | 20 | 21 | 22 |  |
| LDL_H | n | 1 | 18 | 76 | 4 | 0 | 99 |
|  | % | 1.0 | 18.2 | 76.8 | 4.0 | 0.0 | 100 |
| LDL_L | n | 0 | 22 | 73 | 5 | 1 | 101 |
|  | % | 0.0 | 21.8 | 72.3 | 5.0 | 1.0 | 100 |

P=0.8

Table S13

|  |  | DYS 456 |  |  |  |  |  | Total |
| --- | --- | --- | --- | --- | --- | --- | --- | --- |
|  |  | 13 | 14 | 15 | 16 | 17 | 18 |  |
| BP_H | n | 0 | 9 | 31 | 44 | 13 | 3 | 100 |
|  | % | 0.0 | 9.0 | 31.0 | 44.0 | 13.0 | 3.0 | 100 |
| BP_L | n | 2 | 8 | 28 | 46 | 16 | 2 | 102 |
|  | % | 2.0 | 7.8 | 27.5 | 45.1 | 15.7 | 2.0 | 100 |

P=0.8

Table S14

|  |  | DYS 389I |  |  |  | Total |
| --- | --- | --- | --- | --- | --- | --- |
|  |  | 12 | 13 | 14 | 15 |  |
| BP_H | n | 10 | 74 | 15 | 0 | 99 |
|  | % | 10.1 | 74.7 | 15.2 | 0.0 | 100 |
| BP_L | n | 16 | 76 | 9 | 1 | 102 |
|  | % | 15.7 | 74.5 | 8.8 | 1.0 | 100 |

P=0.3

Table S15

|  |  | DYS 389II |  |  |  |  |  | Total |
| --- | --- | --- | --- | --- | --- | --- | --- | --- |
|  |  | 28 | 29 | 30 | 31 | 32 | 33 |  |
| BP_H | n | 9 | 27 | 41 | 16 | 3 | 1 | 97 |
|  | % | 9.3 | 27.8 | 42.3 | 16.5 | 3.1 | 1.0 | 100 |
| BP_L | n | 7 | 26 | 50 | 17 | 0 | 0 | 100 |
|  | % | 7.0 | 26.0 | 50.0 | 17.0 | 0.0 | 0.0 | 100 |

P=0.45

Table S16

|  |  | DYS 19 |  |  |  |  | Total |
| --- | --- | --- | --- | --- | --- | --- | --- |
|  |  | 13 | 14 | 15 | 16 | 17 |  |
| BP_H | n | 3 | 21 | 23 | 30 | 20 | 97 |
|  | % | 3.1 | 21.6 | 23.7 | 30.9 | 20.6 | 100 |
| BP_L | n | 6 | 19 | 18 | 33 | 25 | 101 |
|  | % | 5.9 | 18.8 | 17.8 | 32.7 | 24.8 | 100 |

P=0.7

Table S17

|  |  | DYS 385 I |  |  |  |  |  |  |  | Total |
| --- | --- | --- | --- | --- | --- | --- | --- | --- | --- | --- |
|  |  | 10 | 11 | 12 | 13 | 14 | 15 | 16 | 17 |  |
| BP_H | n | 16 | 49 | 10 | 3 | 13 | 4 | 2 | 0 | 97 |
|  | % | 16.5 | 50.5 | 10.3 | 3.1 | 13.4 | 4.1 | 2.1 | 0.0 | 100 |
| BP_L | n | 20 | 48 | 6 | 7 | 12 | 1 | 4 | 2 | 100 |
|  | % | 20.0 | 48.0 | 6.0 | 7.0 | 12.0 | 1.0 | 4.0 | 2.0 | 100 |

P=0.4

Table S18

|  |  | DYS 391 |  |  |  | Total |
| --- | --- | --- | --- | --- | --- | --- |
|  |  | 9 | 10 | 11 | 12 |  |
| BP_H | n | 3 | 52 | 42 | 2 | 99 |
|  | % | 3.0 | 52.5 | 42.4 | 2.0 | 100 |
| BP_L | n | 0 | 56 | 44 | 2 | 102 |
|  | % | 0.0 | 54.9 | 43.1 | 2.0 | 100 |

P=0.4

Table S19

|  |  | DYS 439 | |  |  |  | Total |
| --- | --- | --- | --- | --- | --- | --- | --- |
|  |  | 10 | 11 | 12 | 13 | 14 |  |
| BP_H | n | 33 | 38 | 18 | 8 | 1 | 98 |
|  | % | 33.7 | 38.8 | 18.4 | 8.2 | 1.0 | 100 |
| BP_L | n | 26 | 43 | 27 | 3 | 1 | 100 |
|  | % | 26.0 | 43.0 | 27.0 | 3.0 | 1.0 | 100 |

P=0.2

Table S20

|  |  | DYS 635 |  |  |  |  |  | Total |
| --- | --- | --- | --- | --- | --- | --- | --- | --- |
|  |  | 20 | 21 | 22 | 23 | 24 | 25 |  |
| BP_H | n | 1 | 7 | 12 | 64 | 12 | 2 | 98 |
|  | % | 1.0 | 7.1 | 12.2 | 65.3 | 12.2 | 2.0 | 100 |
| BP_L | n | 3 | 9 | 11 | 68 | 11 | 1 | 103 |
|  | % | 2.9 | 8.7 | 10.7 | 66.0 | 10.7 | 1.0 | 100 |

P=0.9

Table S21

|  |  | GATA H4 |  |  |  | Total |
| --- | --- | --- | --- | --- | --- | --- |
|  |  | 10 | 11 | 12 | 13 |  |
| BP_H | n | 1 | 20 | 60 | 15 | 96 |
|  | % | 1.0 | 20.8 | 62.5 | 15.6 | 100 |
| BP_L | n | 2 | 24 | 60 | 14 | 100 |
|  | % | 2.0 | 24.0 | 60.0 | 14.0 | 100 |

P=0.9

Table S22

|  |  | DYS 437 |  |  | Total |
| --- | --- | --- | --- | --- | --- |
|  |  | 14 | 15 | 16 |  |
| BP_H | n | 69 | 20 | 10 | 99 |
|  | % | 69.7 | 20.2 | 10.1 | 100 |
| BP_L | n | 72 | 20 | 11 | 103 |
|  | % | 69.9 | 19.4 | 10.7 | 100 |

P=0.9

Table S23

|  |  | DYS 438 |  |  |  |  | Total |
| --- | --- | --- | --- | --- | --- | --- | --- |
|  |  | 9 | 10 | 11 | 12 | 13 |  |
| BP_H | n | 5 | 22 | 51 | 19 | 0 | 97 |
|  | % | 5.2 | 22.7 | 52.6 | 19.6 | 0.0 | 100 |
| BP_L | n | 3 | 26 | 59 | 14 | 1 | 103 |
|  | % | 2.9 | 25.2 | 57.3 | 13.6 | 1.0 | 100 |

P=0.6

Table S24

|  |  | DYS 448 |  |  |  |  |  | Total |
| --- | --- | --- | --- | --- | --- | --- | --- | --- |
|  |  | 17 | 18 | 19 | 20 | 21 | 22 |  |
| BP_H | n | 0 | 1 | 16 | 73 | 6 | 2 | 98 |
|  | % | 0.0 | 1.0 | 16.3 | 74.5 | 6.1 | 2.0 | 100 |
| BP_L | n | 1 | 3 | 19 | 71 | 7 | 0 | 101 |
|  | % | 1.0 | 3.0 | 18.8 | 70.3 | 6.9 | 0.0 | 100 |

P=0.5
